# Parallel Behind Your Head

**DOI:** 10.1177/2041669518781141

**Published:** 2018-06-13

**Authors:** Astrid M. L. Kappers, Aytun Ö. R. Çetinkaya, Giulio S. Tan

**Affiliations:** VU Amsterdam, the Netherlands

**Keywords:** haptics/touch, frames of reference, haptic space, parallel

## Abstract

A miniature hair clip set-up presented to the first author gave inspiration for this study. After a number of studies investigating what is haptically perceived as parallel on horizontal, frontoparallel or midsagittal planes, the present study focusses on what is felt as parallel behind your head. The results show convincingly that also in this condition physically parallel is not the same as haptically parallel. Moreover, the deviations are large, idiosyncratic and in a direction predicted by assuming a biasing influence of an egocentric reference frame.

In 2005, the first author was promoted to full professor at Utrecht University. Her group decided that this was an excellent occasion to give her an appropriate present: a hair clip. This hair clip was carefully selected and personalized by decorating it with a miniature version of a set-up that she used in her parallel setting experiments (e.g., Kappers, 1999; Kappers & Koenderink 1999). Somewhat to everyone’s surprise, also parallel settings on this hair clip turned out to be far from physically parallel. The hair clip with a parallel setting of the first author can be seen in Figure 1. Already at that time, the plan emerged to investigate ‘parallel behind your head’ in more detail, but only recently we actually ran such an experiment.

**Figure 1.**
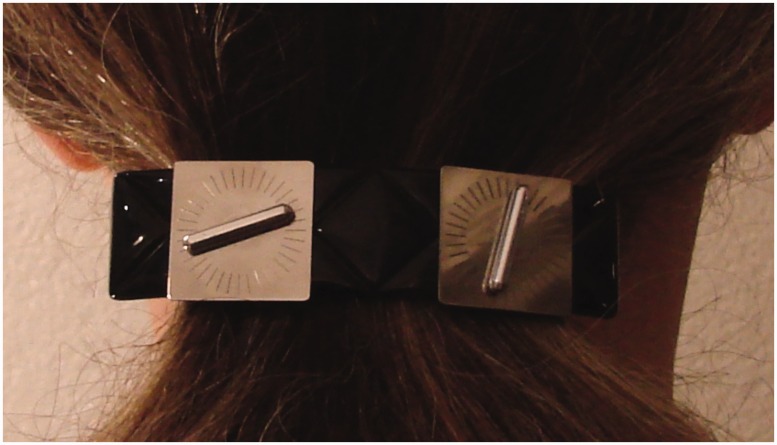
The hair clip that served as inspiration for the current experiment. The setting shows what the first author perceives as parallel.

The idea for the experiments carried out in 1999 arose after reading a paper by Blumenfeld (1937) in which he showed that the parallel lines haptically constructed by blindfolded participants were far from straight. A horizontal set-up was created on which a reference bar could be placed in a certain orientation, and a test bar at another location had to be rotated in such a way that it felt parallel to this reference bar. Both in unimanual and bimanual experiments, large but systematic and idiosyncratic deviations were found: A test bar located to the right of a reference bar has to be rotated clockwise in order to be felt as parallel (Kappers, 1999; Kappers & Koenderink, 1999). In a study with 68 participants, the deviations ranged from 8.0° to 90.5° with an average of 41.3° (Kappers, 2003). Such systematic deviations have been confirmed and investigated by independent other researchers (e.g., Fernández-Díaz & Travieso 2011; Kaas & Van Mier 2006; Van Mier, 2013). The current most plausible explanation for these deviations is that the parallel settings are caused by the biasing influence of an egocentric reference frame, which is probably a combination of a hand and body reference frame. A compelling influence of the hand reference frame is shown by Kappers and Liefers (2012).

For the current experiment, two new set-ups similar to the hair clip were created. This was necessary as not all potential participants had sufficient hair to which a hair clip could be attached. A circuit board plotter (LPKF ProtoMat C60) was used to engrave two circles with tick marks every 6° (i.e., 1 minute) on plastic plates. On one plate, the distance between the centres of the circles was 5 cm, and on the other, it was 13 cm. Small rotatable pointed bars were placed in the centres of the circles. The plastic plates were fixed to a stand and they could be adjusted in height (see Figure 2).

**Figure 2.**
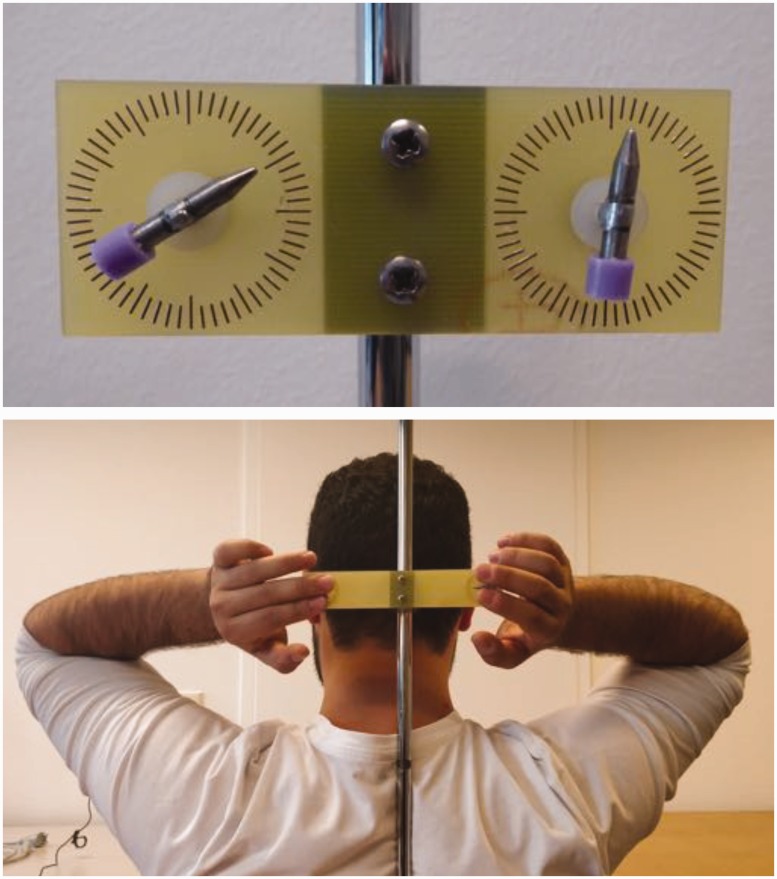
Experimental set-ups. Top: the set-up with a 5-cm distance between the bars. The bars show an example of what on average is set as parallel. Bottom: participant making a setting on the set-up with a 13-cm distance.

The experiment fell within the research program approved by the Ethical Committee of the Department of Human Movement Sciences. The 50 participants (equal numbers of males and females, age range: 18–65 years) contributed voluntarily, and they provided written informed consent. They seated themselves with their head against the set-up (see Figure 2). Reference orientations were 10, 25, 45 and 55 minutes, which were presented once on the left side and once on the right side, on both set-ups. This resulted in 16 bimanual trials for each participant. The maximum time allowed for a trial was 10 seconds. Participants were free in their choice of strategy; in practice, some used their whole hands, others preferred holding the bars between their fingers.

The settings of the participants were noted down in minutes, but for comparison with previous studies, the deviations were transformed to degrees (1 minute equals 6°). The deviations are defined as ‘orientation of the right bar’ minus the ‘orientation of the left bar’, irrespective of whether the bar is a reference bar or a test bar (note that this definition will result in a sign opposite to that of the definition used in most earlier studies). The results can be seen in Figure 3. For the 5-cm set-up, the average deviations ranged from 16.5° to 84.8°, with an average of 52.6°. For the 13-cm set-up, the average deviations ranged from −13.5° to 87°, with an average of 53°. There is a significant correlation between the settings in the two set-ups (*r* = .7, *p* = .000). Non-parametric Mann–Whitney Wilcoxon tests showed that there was no difference between the deviations obtained with the two set-ups or between the average deviations of males (55°) and females (50°) (see also Figure 3).

**Figure 3.**
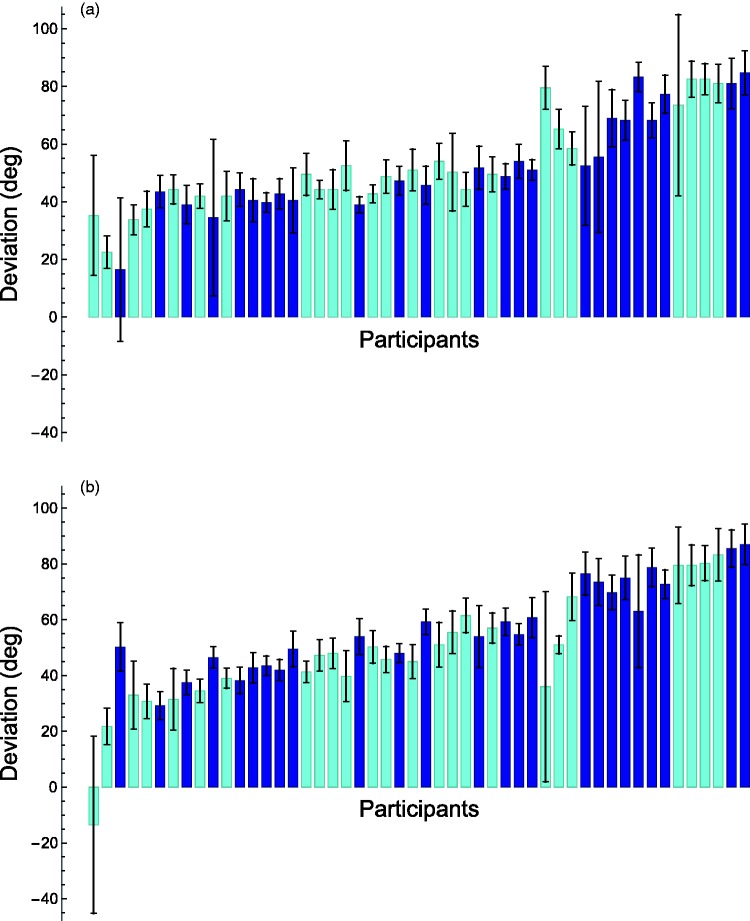
Individual averaged deviations for the two experimental conditions. (a) Set-up 5-cm distance. (b) Set-up 13-cm distance. Error bars indicate standard errors. Participants are ordered according to their average deviation in the two conditions and this is thus the same for (a) and (b). Light bars (cyan) belong to female participants and dark bars (blue) to male participants.

It can clearly be seen that also in hair clip set-ups, large, systematic and idiosyncratic deviations are found. Although the hand and arm orientations of the participants in the current experiment have not been monitored systematically, Figure 2 gives a good indication of how the hands are typically rotated towards each other. These hand/arm orientations are very similar to those in condition 180° of Kappers and Liefers (2012), albeit that in that study the hands were in front of the body. Also the sizes of the obtained deviations are quite comparable (52° in the 180° condition). In Kappers and Viergever (2006), the hand orientations were also systematically varied. In terms of hand orientations, the convergent condition in that study comes closest to the present experiment, but the obtained deviations were not significantly different from 0. The critical difference between these experiments is that here the hands were located at more or less the same place, whereas in the 2006 study, the hands were 60 cm apart. In that situation, not only a hand-centred reference frame influenced the settings, also a body reference frame had a substantial influence. As in that particular situation, these reference frames worked in opposite directions, the deviations were minimized.

Whereas most previous studies (e.g., Kappers, 2003; Van Mier, 2013) found significantly larger deviations with female participants, the current study did not. Interestingly, the only other study in which the hands were also placed at more or less the same location, namely that of Kappers and Liefers (2012), also did not find any difference. We do not yet have an explanation for this difference.

In conclusion, we can say that the positive deviations obtained with the hair clip set-ups are fully consistent with a biasing influence of an egocentric reference frame and with the results of previous studies.
